# Anti-Gouty Arthritis and Anti-Hyperuricemia Properties of *Sanghuangporus vaninii* and *Inonotus hispidus* in Rodent Models

**DOI:** 10.3390/nu14204421

**Published:** 2022-10-21

**Authors:** Zhen Sun, Zhige Li, Yunyun Tan, Xiuxiu Wang, Chunxia Wang, Mingyuan Dong, Honghan Liu, Heng Chen, Yu Li, Lanzhou Li, Di Wang

**Affiliations:** 1 Engineering Research Center of Chinese Ministry of Education for Edible and Medicinal Fungi, Jilin Agricultural University, Changchun 130118, China; 2 School of Life Sciences, Jilin University, Changchun 130012, China; 3 Joint International Research Laboratory of Modern Agricultural Technology, Ministry of Education, Jilin Agricultural University, Changchun 130118, China

**Keywords:** *Sanghuangporus vaninii*, *Inonotus hispidus*, hyperuricemia, gouty arthritis, inflammation

## Abstract

Acute inflammation and hyperuricemia are associated with gouty arthritis. As an edible and therapeutic mushroom, *Sanghuangporus vaninii* (SV) has an inhibitory effect on tumorigenesis, and *Inonotus hispidus* (IH) exhibits anti-tumor, anti-inflammatory, and antioxidant properties. In this study, uric acid (UA) and xanthine oxidase (XOD) levels in hyperuricemic mice were examined to determine the regulatory effects of SV and IH. SV and IH reversed the pathogenic state of elevated UA levels in the serum and reduced levels of XOD in the serum and liver of mice with hyperuricemia. SV and IH affected the inflammatory response in rats with acute gouty arthritis. Compared to vehicle-treated rats, monosodium urate crystals (MSU) increased the swelling ratio of the right ankle joints. SV and IH administration significantly reduced swelling and inflammatory cell infiltration. SV reduced the levels of interleukin-8 (IL-8) and chemokine ligand-2 (CCL-2), whereas IH reduced the levels of matrix metalloproteinase-9 (MMP-9), CCL-2, and tumor necrosis factor-α (TNF-α), which were confirmed in articular soft tissues by immunohistochemistry. In summary, our data provide experimental evidence for the applicability of SV and IH in gouty arthritis and hyperuricemia treatment.

## 1. Introduction

Gouty arthritis is characterized by recurrent arthritic episodes caused by the deposition of monosodium urate crystals (MSU) [1], which are associated with various diseases including arterial hypertension [2], type 2 diabetes mellitus [3], and cardiovascular disease [4]. 

As the end product of purine metabolism, the overproduction of uric acid (UA) is responsible for the occurrence of hyperuricemia [5]. Xanthine oxidase (XOD) plays an irreplaceable role in UA accumulation and reactive oxygen species (ROS) generation [6]. Hyperuricemia is accompanied by MSU deposition in the joints or surrounding tissues, leading to gouty arthritis [7]. At the onset of gouty arthritis, MSU is involved in caspase-1 activation, which causes the production of interleukin-1β (IL-1β) and interleukin-18 (IL-18), inducing proinflammatory responses [8]. The main inflammatory cells in gouty arthritis attacks are mononuclear macrophages and multinucleated macrophages, which produce various cytokines such as tumor necrosis factor-α (TNF-α). Matrix metalloproteinase-9 (MMP-9), influenced by TNF-α, leads to matrix degradation in gouty arthritis tophi, which is helpful for the infiltration of inflammatory cells in the affected area [9]. In recent studies, yeast extract powder (YEP) and potassium oxonate (OXO) were used to establish a mouse model with a high level of UA to simulate human hyperuricemia, where YEP modifies the normal purine metabolism to produce a considerable quantity of UA, and OXO inhibits the UA oxidase activity and uricolysis of UA [10]. The gout model is reproduced in rats by administering exogenous MSU crystals to create a state expressing a lot of proinflammatory cytokines, which resembles a gout attack in a human [11]. The hyperuricemia mice model induced by YEP and OXO as well as the acute gouty arthritis rat model induced by MSU have been used to evaluate the anti-hyperuricemia and anti-gouty arthritis properties of some natural products such as sunflower head extract and celery seed extracts [12,13]. 

As a clinical drug for gouty arthritis treatment, colchicine (COL) can suppress the activation of caspase-1 and prevent the release of IL-1β and IL-18, thereby inhibiting the occurrence of gouty arthritis [8]. However, its excessive use may cause gastrointestinal discomfort, diarrhea, and kidney problems, increase the risk of heart disease, and cause other side effects [14]. Although nonsteroidal anti-inflammatory drugs can also be used for gouty arthritis treatment, they always cause gastrointestinal and cardiovascular problems [15]. Allopurinol (AL), an XOD inhibitor, is the most prescribed therapeutic agent for hyperuricemia and is associated with allergic reactions including hepatotoxicity and generalized rash manifestations [16].

Therefore, a safe and effective agent for relieving gout symptoms is urgently needed. Fungi have long been used to treat a wide range of diseases because of their various bioactive effects [17] including antioxidative and anti-inflammatory properties [18,19]. According to a report, *Ganoderma applanatum* can reduce the absorption of purine in the gastrointestinal tract by downregulating the level of nucleoside transporter 2 protein in the gastrointestinal tract of mice with hyperuricemia, resulting in a significant hypouric acid effect [20]. *Inonotus obliquus* inhibits XOD activity in the serum and liver and urate transporter 1 to treat hyperuricemia [21]. *Sanghuang* is a polypore belonging to Hymenochaetaceae in Basidiomycota and has been intensively studied over the past few decades [22]. In China, several studies have focused on *Tropicoporus linteus* [23]. According to previous research, *Phellinus igniarius* can reduce the degree of ankle swelling and shows obvious anti-inflammatory activity by inhibiting the expression of intercellular adhesion molecule-1 (ICAM-1), IL-1β, and IL-6 in a rat model of acute gouty arthritis [24]. *Sanghuangporus vaninii* (SV), which is mainly parasitic on deciduous trees such as ash and mulberry, has been confirmed to inhibit tumorigenesis [25]. *Inonotus hispidus* (IH) exhibits anti-oxidant, anti-inflammation, and anti-tumor properties [26,27,28]. However, the anti-gouty arthritis effects of SV and IH have not yet been systemically reported.

In this study, the anti-hyperuricemia and anti-gouty arthritis effects of both SV and IH were investigated in animal models. Further data suggest that these effects are related to their anti-oxidative stress and anti-inflammatory effects by inhibiting the formation of XOD. These data provide evidence for the potential application of SV and IH for gouty arthritis treatment.

## 2. Materials and Methods

### 2.1. Experiments Performed on Hyperuricemia Induced by YEP and OXO in Mice

Thirty male BALB/c mice (8-week-old, 20–22 g) were purchased from Liaoning Changsheng Biotechnology Co., Ltd. (Benxi, China) (SCXK (Liao) 2022-0001) and housed in ventilated cages under standard conditions: ambient temperature of 22–24 °C, relative humidity of 55%, with a light/dark cycle of 12 h (lights on 7:00 a.m.–7:00 p.m.). All mice were provided ad libitum access to food and water. After a week of acclimation, the mice were randomly divided into five groups (*n* = 6) including the Ctrl group, model group, 20 mg/kg of AL (Hefei Jiulian Pharmaceutical Co., Ltd., Hefei, China) treated group, 1.5 g/kg SV treated group, and 1.5 g/kg IH treated groups. The Ctrl and model mice were orally administered D.D. water, whereas the other mice were orally administered AL, SV, or IH for 8 days. From the first to eighth days, 20 g/kg YEP was administered by gavage to all mice except the Ctrl mice, which were orally administered D.D. water. On the sixth to eighth days, mice were intraperitoneally injected with 300 mg/kg OXO, except for the Ctrl mice. One hour after the last administration, blood was collected from the caudal veins of mice. Thereafter, the mice were euthanized by carbon dioxide inhalation, and the liver, spleen, and kidney tissues were removed rapidly. Part of the tissues was flash-frozen and stored at −80 °C until analysis, and the remaining tissues were fixed in 10% neutral buffered formalin solution. All animal procedures were approved and implemented in accordance with the Institutional Animal Ethics Committee of Jilin University (permit ID: SY202108004 and SY202106006).

### 2.2. Experiments Performed on Acute Gouty Arthritis Induced by MSU Crystal Injection in Rats

Thirty male Wistar rats (8-week-old, 320–390 g), purchased from Liaoning Changsheng Biotechnology Co., Ltd. (Benxi, China) (SCXK (Liao) 2022-0001), were housed in ventilated cages under standard conditions: ambient temperature of 22–24 °C, relative humidity of 55%, and a light/dark cycle of 12 h (lights on 7:00 a.m.–7:00 p.m.). All rats were provided ad libitum access to food and water. After a week of acclimation, the rats were randomly divided into five groups (*n* = 6) including the Ctrl group, model group, 0.3 mg/kg COL (Xishuangbanna Banna Pharmaceutical Co., Ltd., Jinghong, China) treated group, 1.5 g/kg SV, and 1.5 g/kg IH treated groups. The Ctrl and model mice were orally administered D.D. water, whereas the other mice were orally administered COL, SV, or IH for 8 days. On the sixth day, 3 mg MSU dissolved in normal saline was injected into the synovial space of the right ankle in all mice, except the Ctrl mice, which were injected with the same amount of normal saline. On the sixth to eighth days, the ankle circumference was measured using Vernier calipers at 0, 4, 8, 12, 24, and 48 h after MSU injection. The swelling rate of the ankle joints was calculated using the following formula:

Swelling ratio (%) = (C_t_ − C_0_)/C_0_ × 100%

where C_t_ represents the corresponding perimeter at t h, and C_0_ represents the initial perimeter at 0 h. After 48 h, blood was collected from the caudal veins of the rats. The rats were euthanized by carbon dioxide inhalation, and the liver, spleen, and kidney tissues were removed rapidly. Part of the tissues was flash-frozen and stored at −80 °C until analysis, and the remaining tissues were fixed in 10% neutral buffered formalin solution. The right ankle joints of the rats were collected, fixed in 4% paraformaldehyde in phosphate buffer solution (PBS), and decalcified with Ethylene Diamine Tetraacetic Acid (EDTA) (G1105, Servicebio, Wuhan, China). All animal procedures were approved and implemented in accordance with the Institutional Animal Ethics Committee of Jilin University (permit ID: SY202108003).

### 2.3. Hematoxylin and Eosin (H&E) Staining

The fixed tissues including the liver, spleen, and kidney of mice and rats, and the ankle joints of rats were dehydrated using different concentrations of alcohol and xylene, dipped in wax, and then embedded in paraffin for sectioning with a thickness of approximately 4 μm. After staining the sections with hematoxylin dye solution for 3–5 min and eosin dye solution for 5 min, dye solution for 5 min, absolute ethanol, and xylene were added for dehydration and transparency, and the slices were sealed with neutral gum as described in our previous study [29]. Histological sections were observed and photographed under a light microscope (40×, 100× and 200×) (Olympus Corporation, Tokyo, Japan).

### 2.4. Biochemical Tests

In rats with acute gouty arthritis, the serum levels of interleukin-8 (IL-8, ml002885), MMP-9 (ml003043), chemokine ligand-2 (CCL-2, ml002960), and TNF-α (ml002859) were determined using enzyme-linked immunosorbent assay (ELISA) kits (Shanghai Enzyme-linked Biotechnology Co., Ltd., Shanghai, China), according to the manufacturer’s instructions.

In mice with hyperuricemia, serum UA levels were detected by the enzymatic-colorimetric method using a standard diagnostic kit (C012-2-1, Nanjing Jiancheng Bioengineering Institute, Nanjing, China). The XOD levels in the serum and liver were detected by a colorimetric method using a standard diagnostic kit (A002-1-1, Nanjing Jiancheng Bioengineering Institute, Nanjing, China). All of the testing procedures were performed according to the manufacturer’s instructions.

### 2.5. Immunohistochemistry (IHC) Analysis

The rats’ ankle joint paraffin sections were dewaxed with xylene, rehydrated with different concentrations of alcohol, placed in pepsin antigen repair solution (G0142) (Servicebio, Wuhan, China) for antigen repair, and then washed three times in PBS buffer (G0002) (Servicebio, Wuhan, China) for 5 min. The sections were placed in a 3% hydrogen peroxide solution (Sinopharm, Beijing, China) for 25 min to block endogenous peroxidase and washed. After blocking with 3% bovine albumin (G5001) (Servicebio, Wuhan, China) for 30 min, the primary antibodies CCL-2 (GB11199, 1:200 dilution) (Servicebio, Wuhan, China), MMP-9 (GB12132, 1:500 dilution) (Servicebio, Wuhan, China), IL-8 (A00423-1, 1:500 dilution) (Boster, Wuhan, China), TNF-α (GB11188, 1:100 dilution) (Servicebio, Wuhan, China), and the corresponding secondary antibody HRP-goat anti-rabbit (GB23303, 1:200 dilution) (Servicebio, Wuhan, China) were added and incubated for 10 h and 50 min at 4 °C, respectively. A diaminobenzidine (G1212, Servicebio, Wuhan, China) working solution was used for color development for approximately 10 min. After hematoxylin staining, dehydration using xylene and other reagents, and sealing with neutral gum, the histological sections were observed and photographed under a light microscope (100×) (Olympus Corporation, Tokyo, Japan). The percentage of positive areas in the 12 non-overlapping fields was averaged for each group.

### 2.6. Western Blot Analysis

The total liver protein was extracted using radioimmunoprecipitation assay (RIPA) buffer (PC101, EpiZyme, Shanghai, China) containing 1% protease and phosphatase inhibitor (P002, New Cell & Molecular Biotech Co., Ltd., Wuxi, China) using a high-throughput tissue grinder (SCIENTZ-48, Ningbo Scientz Biotechnology Co., Ltd., Ningbo, China). The liver proteins (40 μg) were analyzed using a bicinchoninic acid protein assay kit (Merck, Darmstadt, Germany), separated by 10% sodium dodecyl sulfate-polyacrylamide gel electrophoresis (SDS-PAGE) (PG112, Shanghai Epizyme Biomedical Technology Co., Ltd., Shanghai, China), and then blotted onto a 0.45 μm polyvinylidene difluoride (PVDF) membrane (10600023, Cytiva, Marlborough, MA, USA). Rapid closure solution (P30500, New Cell & Molecular Biotech, Wuxi, China) was used to block the membranes, and then the primary antibodies XOD (AB109235, 1:1000 dilution) (Abcam, MA, USA) and glyceraldehyde-3-phosphate dehydrogenase (GAPDH) (e-ab-20032, 1:1000 dilution) (Elabscience, Wuhan, China) were added and incubated at 4 °C overnight. After washing with 0.1% Tween-20 Tris buffer, the corresponding secondary antibodies, horseradish peroxidase-conjugated goat anti-mouse IgG (e-ab-1001, 1:1000 dilution) (Elabscience, Wuhan, China) and goat anti-rabbit IgG (e-ab-1003, 1:1000 dilution) (Elabscience, Wuhan, China), were added and incubated for 4 h at 4 °C. Protein bands were visualized using an enhanced chemiluminescence detection system (Tanon 5200, Tanon Science & Technology Co., Ltd., Shanghai, China) and ultra-high-sensitivity enhanced chemiluminescence (ECL) kits (GK10008, GLPBIO, Montclair, CA, USA). Protein expression levels were measured using ImageJ software (National Institutes of Health, Bethesda, MD, USA) and normalized to GAPDH. 

### 2.7. Statistics

All values are presented as the mean ± SD. Statistical analyses were performed using GraphPad Prism 8 software with one-way analysis of variance (ANOVA) followed by Dunnett’s *t*-test for separate comparisons. *p* < 0.05 was considered to be statistically significant. 

## 3. Results

### 3.1. SV and IH Relieve the Symptoms in Hyperuricemia in Mice

**Figure 1:**
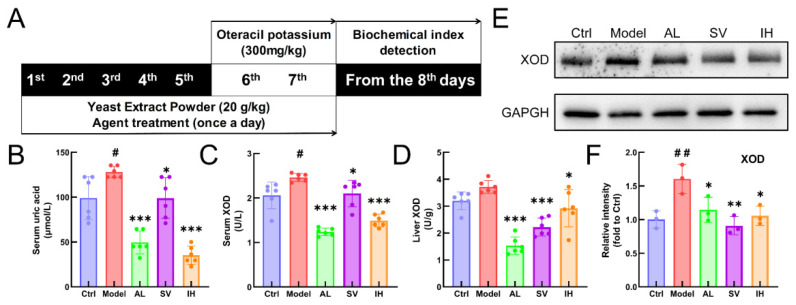
SV and IH relief symptoms on YEP/OXO-induced hyperuricemia in mice. (**A**) Schematic diagram of the experimental schedule performed in the mice with hyperuricemia. In the YEP/OXO-induced mice with hyperuricemia, SV and IH reduced the high serum levels of UA (**B**) and XOD (**C**) and suppressed the levels of XOD (**D**) in the liver. (**E**,**F**) SV and IH reduced the expressions of XOD in the liver of mice with hyperuricemia. Data are shown as means ± SD (*n* = 6 mice/group). ^#^ *p* < 0.05, ^##^ *p* < 0.01 vs. Ctrl group, * *p* < 0.05, ** *p* < 0.01, *** *p* < 0.001 vs. model group.

**Figure 2:**
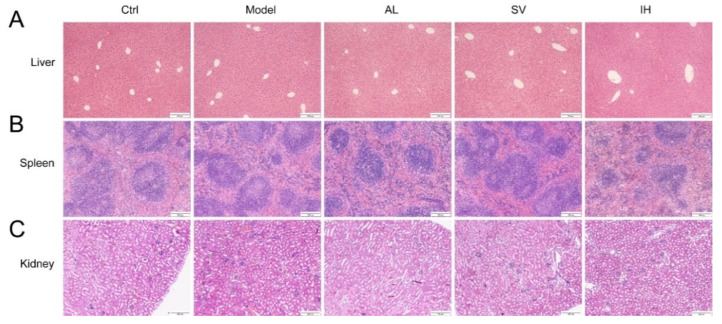
Histopathological assessment of the liver (**A**), spleen (**B**), and kidney (**C**) in mice with hyperuricemia via H&E staining observed with light microscopy (100×). Scale bar, 200 μm.

YEP and OXO administration caused a 29.35% increase in the serum UA levels in mice (*p* < 0.05, Figure 1B), which was reduced by AL up to 61.34% (*p* < 0.001), by SV up to 22.69% (*p* < 0.05), and by IH up to 72.45% (*p* < 0.001) (Figure 1B). AL, SV, and IH failed to influence the body weights and organ indices of mice with hyperuricemia (Table 1). Among all of the experimental mice, no significant changes were observed in the pathological sections including the liver, spleen, and kidney (Figure 2).

Compared with the Ctrl mice, a 19.71% enhancement in serum XOD levels (*p* < 0.05, Figure 1C) was observed in mice with hyperuricemia, which was suppressed by AL up to 49.70% (*p* < 0.001), by SV up to 14.63% (*p* < 0.05), and by IH up to 39.39% (*p* < 0.001) (Figure 1C). Similarly, compared to the vehicle-treated mice with hyperuricemia, AL, SV, and IH resulted in 58.81% (*p* < 0.001), 40.23% (*p* < 0.001), and 21.31% (*p* < 0.05) (Figure 1D) decrements on hepatic XOD, respectively. These results were further confirmed by Western blotting, which showed that the low expression levels of XOD in the liver were in the AL-, SV-, and IH-treated mice with hyperuricemia (*p* < 0.05, Figure 1E,F).

### 3.2. SV and IH Relieve the Symptoms in Rats with Acute Gouty Arthritis

**Figure 3:**
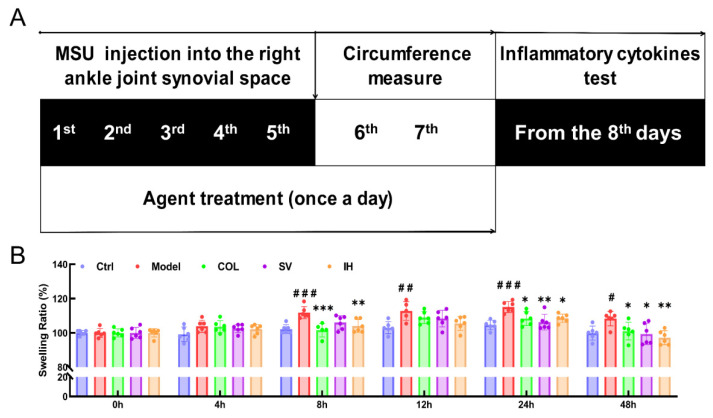
SV and IH relieve the swelling ration of ankle joints in MSU-induced acute gouty arthritis in rats. (**A**) Schematic diagram of the experimental schedule performed in rats with gouty arthritis. (**B**) SV and IH suppressed the swelling ratio of ankle joints in rats with acute gouty arthritis. Data are shown as means ± SD (*n* = 6 rats/group). ^#^ *p* < 0.05, ^##^ *p* < 0.01, ^###^ *p* < 0.001 vs. Ctrl group, * *p* < 0.05, ** *p* < 0.01, *** *p* < 0.001 vs. model group.

**Figure 4:**
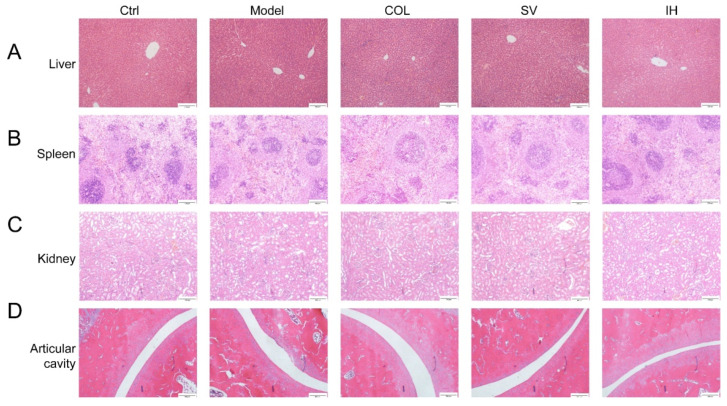
Histopathological assessment of the liver (**A**), spleen (**B**), kidney (**C**), and ankle articular cavity (**D**) in rats with acute gouty arthritis via H&E staining observed with light microscopy (*n* = 3) (100×). Scale bar, 200 μm.

Compared to the Ctrl rats, MSU significantly enhanced the swelling ratio of the right ankle joints of rats from 8 to 48 h (*p* < 0.05, Figure 3B), and this swelling was significantly suppressed by COL administration (*p* < 0.05, Figure 3B). Similarly, SV suppressed swelling at 24 h (*p* < 0.01) and 48 h (*p* < 0.05) (Figure 3B); meanwhile, IH suppressed swelling at 8 h (*p* < 0.01), 24 h (*p* < 0.05), and 48 h (*p* < 0.01) (Figure 3B) after MSU injection. COL, SV, and IH failed to influence the body weights and organ indices of rats with acute gouty arthritis (Table 2). No significant changes were observed in all experimental rats and pathological sections including the liver, spleen, and kidney (Figure 4). 

### 3.3. SV and IH Display the Anti-Inflammatory Effects in Acute Gouty Arthritis

**Figure 5:**
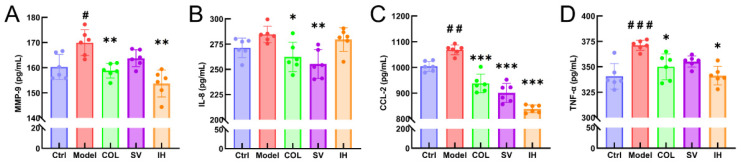
SV and IH inhibited the inflammatory response in acute gouty arthritis rats. The effects of SV and IH on the serum levels of MMP-9 (**A**), IL-8 (**B**), CCL-2 (**C**), and TNF-α (**D**) in acute gouty arthritis rats. Data are shown as means ± SD (*n* = 6 rats/group). ^#^ *p* < 0.05, ^##^ *p* < 0.01, ^###^ *p* < 0.001 vs. Ctrl group, * *p* < 0.05, ** *p* < 0.01, *** *p* < 0.001 vs. model group.

**Figure 6:**
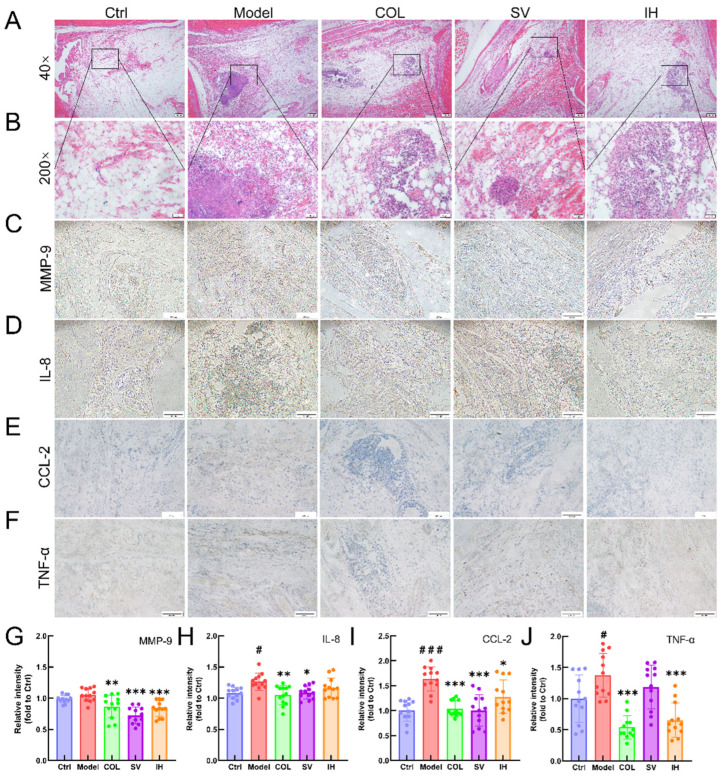
SV and IH showed anti-inflammatory effects in acute gouty arthritis rats. Representative images of H&E-stained pathological sections of ankle articular soft tissues (*n* = 3) (upper panel: 40×, Scale bar, 200 μm) (**A**); (lower panel: 200×, Scale bar, 50 μm) (**B**). Representative images of immunohistochemistry of MMP-9 (**C**), IL-8 (**D**), CCL-2 (**E**), and TNF-α (**F**) in the ankle articular soft tissues of rats (100×, Scale bar, 200 μm). Ratios of the integrated option density to area of the aim protein were measured to quantify the MMP-9 (**G**), IL-8 (**H**), CCL-2 (**I**) and TNF-α (**J**) levels in the ankle articular soft tissues of rats. Data are shown as means ± SD (*n* = 12 images/group). ^#^ *p* < 0.05, ^###^ *p* < 0.001 vs. Ctrl group, * *p* < 0.05, ** *p* < 0.01, *** *p* < 0.001 vs. model group.

Compared with vehicle-treated rats with acute gouty arthritis, SV resulted in 10.31% and 15.67% reductions in the levels of IL-8 (*p* < 0.01; Figure 5B) and CCL-2 (*p* < 0.001; Figure 5C), respectively, and IH resulted in 9.54%, 21.49%, and 8.03% reductions in the levels of MMP-9 (*p* < 0.01; Figure 5A), CCL-2 (*p* < 0.001; Figure 5C), and TNF-α (*p* < 0.05; Figure 5D) in serum, respectively. COL strongly suppressed the MMP-9 (*p* < 0.01; Figure 5A), IL-8 (*p* < 0.05; Figure 5B), CCL-2 (*p* < 0.001; Figure 5C), and TNF-α (*p* < 0.05; Figure 5D) levels in the serum of rats with acute gouty arthritis. Histopathological examination showed that MSU injection caused inflammatory cell infiltration in articular soft tissue, which was significantly suppressed by COL, SV, and IH administration (Figure 6A). In rats with acute gouty arthritis, the suppressive effects of COL, SV, and IH on MMP-9 (*p* < 0.01; Figure 6C,G), IL-8 (*p* < 0.05; Figure 6D,H) (except for IH), CCL-2 (*p* < 0.05; Figure 6E,I), and TNF-α (*p* < 0.001; Figure 6F,J) (except for SV) in articular soft tissues were further confirmed by IHC.

## 4. Discussion

In this study, the anti-hyperuricemia effect and the alleviation effect of SV and IH on ankle joint swelling were first confirmed in mice with hyperuricemia and rats with acute gouty arthritis, respectively. In rats with acute gouty arthritis, the anti-inflammatory properties of SV and IH were successfully confirmed. 

Inhibition of XOD, the main enzyme responsible for producing UA, results in a decrease in the UA levels and alleviation of UA accumulation in the joints. AL, an XOD inhibitor, is the first-line therapeutic approach for hyperuricemia. Similarly, both IH and SV strongly suppressed the serum levels of XOD and hepatic expression of XOD in mice with hyperuricemia, suggesting a reduction in the serum UA levels. In contrast, no significant changes in organs including the liver, spleen, and kidney were observed on pathological analysis among all of experimental groups, suggesting the safety of IH and SV.

Gouty arthritis attacks are accompanied by the infiltration of neutrophilic granulocytes, which enhances the release of lysosomal enzymes and inflammatory factors [30]. In this study, inflammatory cell infiltration induced by MSU injection was strongly suppressed by SV and IH administration. Chemokines such as MMP-9 and CCL-2 contribute to the inflammation associated with gouty arthritis [31,32]. Neutrophil granules contain high levels of MMP-9, produced by invading cells of the monocyte/macrophage lineage [33], which can dissolve collagen types I and II and degrade gelatin, an excessive production of which eventually leads to arthritis [34]. By weakening the extracellular matrix covering the joints or inducing inflammatory factors, MMP-9 may worsen arthritis [35,36]. The overexpression of MMP-9 has been noted in the synovial fluid of patients with various arthritic diseases [37,38]. An MMP inhibitor can prevent the destruction of the joints. 

In experimental pulmonary fibrosis, MMP-9 promotes the release of transforming growth factor-β (TGF-β) and TNF-α [39,40], which has been enhanced in rabbits with acute gouty arthritis [41]. TNF-α can enhance the activity of polymorphonuclear elastase and increase the release of IL-1, which is responsible for acute inflammatory reactions. Accordingly, the blockage of TNF-α production in gouty arthritis induced by MSU crystals could significantly inhibit the expression of E-selectin and the recruitment of polymorphonuclear [42], suggesting that TNF-α receptor antagonists may be selected as a therapeutic regimen to control the acute development of gouty arthritis [43]. TNF-α helps produce SR-A, participating in the production of CCL-2 [44,45], which is a key player in rheumatoid arthritis and can stimulate chemotaxis in mononuclear cells [46]. In rats with acute gouty arthritis, IH strongly reduced the serum and joint soft tissue levels of MMP-9, CCL-2, and TNF-α, confirming its anti-inflammatory properties. In contrast to IH, SV strongly suppressed the levels of CCL-2, MMP-9, and IL-8 in the serum and joint soft rats with acute gouty arthritis. IL-8 induces chemotaxis in neutrophils, causing them to migrate toward the site of infection and inflammation [47]. MSU dramatically increases the synthesis of IL-8 mRNA in synovial endothelial cells, which activates and drives neutrophil aggregation, leading to the initiation of acute gouty arthritis inflammation [48].

Some issues still need to be investigated. In our group, we systematically analyzed the ingredients of IH and SV and found that their different constituents may be responsible for their different effects on gouty arthritis. The material basis of the pharmacological activities of IH and SV has been studied in our ongoing experiments [49]. In contrast, although we found that both IH and SV can suppress the levels of XOD and the inflammatory response, which may be directly related to their anti-hyperuricemia and anti-acute gouty arthritis inflammatory properties, respectively, the detailed mechanisms during these effects should be excavated deeply. Finally, based on our present study, it is difficult to determine which has the advantages of anti-hyperuricemia and anti-acute gouty arthritis efficacies. 

## 5. Conclusions

In this study, we first confirmed the anti-hyperuricemia and anti-acute gouty arthritis inflammatory properties of both IH and SV in mice with hyperuricemia and rats with acute gouty arthritis. Our data provide experimental evidence for the applicability of SV and IH in gouty arthritis treatment.

## Figures and Tables

**Table 1:** Effects of SV and IH on body weight and indices of the liver, spleen, and kidney in mice with hyperuricemia.

	Days	Ctrl	Model	AL	SV	IH
Body weight (g)	1	28.07 ± 1.18	27.92 ± 0.96	27.28 ± 0.52	28.17 ± 0.37	27.57 ± 0.87
	2	28.50 ± 1.34	27.43 ± 0.74	27.67 ± 1.39	28.27 ± 0.95	28.47 ± 0.78
	3	29.07 ± 1.11	27.58 ± 0.62	27.67 ± 1.59	28.27 ± 1.22	29.20 ± 1.09
	4	29.23 ± 0.62	27.50 ± 1.14	27.57 ± 1.49	27.88 ± 0.86	29.00 ± 1.23
	5	28.13 ± 0.62	27.17 ± 0.82	27.90 ± 1.15	27.37 ± 1.06	27.83 ± 1.49
	6	27.63 ± 1.21	26.88 ± 0.94	27.10 ± 0.94	27.33 ± 1.15	28.00 ± 0.64
	7	28.58 ± 0.61	26.92 ± 0.89	26.70 ± 1.21	27.13 ± 1.29	27.90 ± 1.82
Organ index (mg/g)	Liver	47.61 ± 2.74	49.25 ± 3.02	49.83 ± 3.32	45.48 ± 2.33	50.45 ± 2.75
	Spleen	3.01 ± 0.26	3.47 ± 0.31	3.75 ± 0.74	3.40 ± 0.43	3.45 ± 0.38
	Kidney	14.95 ± 0.96	13.67 ± 0.82	14.90 ± 0.39	14.78 ± 1.34	15.48 ± 1.24

Data are presented as the mean ± SD. (*n* = 6) and analyzed via a one-way ANOVA test.

**Table 2:** Effects of SV and IH on body weight and indices of the liver, spleen, and kidney in rats with gouty arthritis.

	Days	Ctrl	Model	COL	SV	IH
Body weight (g)	1	355.93 ± 11.71	356.98 ± 14.91	355.42 ± 11.46	359.05 ± 8.57	362.42 ± 4.71
	2	360.67 ± 9.62	364.62 ± 19.08	364.02 ± 12.78	364.98 ± 10.08	367.00 ± 3.65
	3	351.38 ± 16.14	343.80 ± 12.95	346.70 ± 8.21	353.82 ± 13.42	355.07 ± 4.09
	4	366.93 ± 9.17	377.65 ± 7.16	366.52 ± 5.65	371.22 ± 15.13	370.03 ± 12.50
	5	367.45 ± 16.69	376.10 ± 15.67	370.68 ± 13.62	368.8 ± 14.23	364.28 ± 16.72
	6	361.15 ± 16.74	373.70 ± 13.95	362.38 ± 15.43	376.1 ± 12.20	376.95 ± 8.41
	7	370.92 ± 9.67	376.97 ± 7.63	370.35 ± 9.71	378.13 ± 12.63	375.97 ± 13.69
Organ index (mg/g)	Liver	38.05 ± 2.00	39.19 ± 1.77	38.17 ± 1.66	38.59 ± 2.84	39.81 ± 2.19
	Spleen	2.06 ± 0.22	2.07 ± 0.13	2.00 ± 0.21	1.78 ± 0.23	2.46 ± 0.35
	Kidney	6.94 ± 0.77	6.88 ± 0.79	6.71 ± 0.58	6.80 ± 0.55	7.58 ± 0.86

Data are presented as the mean ± SD. (*n* = 6) and analyzed via a one-way ANOVA test.

## Data Availability

Not applicable.
